# Plk4-dependent phosphorylation of STIL is required for centriole duplication

**DOI:** 10.1242/bio.201411023

**Published:** 2015-02-20

**Authors:** Anne-Sophie Kratz, Felix Bärenz, Kai T. Richter, Ingrid Hoffmann

**Affiliations:** Cell Cycle Control and Carcinogenesis, F045, German Cancer Research Center, DKFZ, 69120 Heidelberg, Germany

**Keywords:** Plk4, STIL, Centriole duplication, Phosphorylation, Centrosome

## Abstract

Duplication of centrioles, namely the formation of a procentriole next to the parental centriole, is regulated by the polo-like kinase Plk4. Only a few other proteins, including STIL (SCL/TAL1 interrupting locus, SIL) and Sas-6, are required for the early step of centriole biogenesis. Following Plk4 activation, STIL and Sas-6 accumulate at the cartwheel structure at the initial stage of the centriole assembly process. Here, we show that STIL interacts with Plk4 *in vivo*. A STIL fragment harboring both the coiled-coil domain and the STAN motif shows the strongest binding affinity to Plk4. Furthermore, we find that STIL is phosphorylated by Plk4. We identified Plk4-specific phosphorylation sites within the C-terminal domain of STIL and show that phosphorylation of STIL by Plk4 is required to trigger centriole duplication.

## INTRODUCTION

Centrosomes organize the microtubule cytoskeleton and are essential for the assembly of cilia in animal cells (Brito et al., 2012). They participate in mitotic spindle formation and thereby ensure faithful chromosome segregation during cell division. One centrosome consists of a pair of centrioles surrounded by the pericentriolar material (PCM). Like DNA replication, centrosome duplication is tightly regulated and restricted to only once per cell cycle. Thus, controlling centriole numbers ensures that cells have the proper number of centrosomes and cilia. Failures in centriole duplication result in abnormal centriole numbers, which have been linked to genomic instability and tumorigenesis.

Studies in *C. elegans* reveal an evolutionary conserved pathway in centriole formation (Pelletier et al., 2006; Delattre et al., 2006), and several key components were identified in *Drosophila melanogaster* and human cells (Spd-2/Cep192; Zyg-1/SAK/Plk4; Sas-6; Sas-5/Ana-2/STIL and Sas-4/CPAP) (Brito et al., 2012). A central role in the control of centriole biogenesis has been attributed to Plk4, a member of the polo-like kinase family. Depletion of Plk4 results in a failure to build new centrioles and, conversely, overexpression of Plk4 drives the assembly of excessive numbers of newly formed centrioles (Bettencourt-Dias et al., 2005; Habedanck et al., 2005; Kleylein-Sohn et al., 2007; Peel et al., 2007). Dysregulation of Plk4 has been linked to genomic instability and tumorigenesis that is caused by loss of centrosomal numerical integrity (Basto et al., 2008; Ko et al., 2005; Macmillan et al., 2001; Liu et al., 2012). Intriguingly, Plk4 is a structurally divergent Plk family member as it harbors two tandem polo-boxes (PB1 and PB2) (also called cryptic polo box) and a single carboxy-terminal PB, PB3, which confers homodimerization (Leung et al., 2002). Plk4 protein levels are regulated by the SCF^Slimb/β-TrCP^ ubiquitin ligase that recognizes Plk4 after homodimer-dependent *trans*-autophosphorylation of the phosphodegron known as the downstream regulatory element (DRE) (Guderian et al., 2010; Cunha-Ferreira et al., 2009; Rogers et al., 2009; Brownlee et al., 2011; Holland et al., 2010). This activity is counteracted during M phase by protein phosphatase 2A-dependent dephosphorylation, thereby stabilizing Plk4 and allowing a brief mitotic debut that restricts centriole duplication to a single event per cell cycle (Brownlee et al., 2011). Two centrosomal proteins, Cep152 and Cep192, bind to PB1 and PB2 and collaborate to recruit Plk4 to the centrosome (Cizmecioglu et al., 2010; Hatch et al., 2010; Dzhindzhev et al., 2010; Sonnen et al., 2013; Park et al., 2014). To date, only few substrates of Plk4 have been identified, namely GCP6, a part of the gamma-tubulin ring complex (Bahtz et al., 2012), Fbxw5, a component of the SCF E3 ubiquitin ligase involved in regulation of Sas-6 protein levels (Puklowski et al., 2011), and Cep152 (Hatch et al., 2010).

STIL/SIL (SCL/TAL1 interrupting locus), an ortholog of Ana2/Sas-5, is a centriole duplication factor that colocalizes with Sas-6 to the procentriolar cartwheel region (Tang et al., 2011; Stevens et al., 2010a; Stevens et al., 2010b; Vulprecht et al., 2012; Arquint et al., 2012). Depletion of STIL completely blocks centriole formation. STIL mutations have been identified in microcephaly patients (Papari et al., 2013; Kumar et al., 2009) and cause left-right asymmetry defects in mice and disorganized mitotic spindles in zebrafish (Izraeli et al., 1999; Pfaff et al., 2007). At nuclear envelope breakdown, Cdk1 regulates the translocation of STIL from early mitotic centrosomes to the cytoplasm thus triggering cartwheel disassembly. Cytoplasmic STIL is then degraded by the APC/C-proteasome pathway at mitotic exit (Arquint and Nigg, 2014).

Here, we identify STIL as a novel substrate and interacting partner of Plk4. Prior to centriole duplication STIL starts to colocalize with Plk4 to the centrosome. Furthermore, we found that the polo-box domain including PB1, 2 and 3 of Plk4 is not sufficient to mediate interaction with STIL and that in particular the N-terminal region of Plk4 is required for the interaction. Plk4 strongly binds to a STIL fragment that contains both the coiled-coil (CC) domain and the STAN motif. STIL is phosphorylated by Plk4 at five amino acid residues. Furthermore, we show that Plk4-dependent phosphorylation of STIL regulates centriole duplication.

## MATERIALS AND METHODS

### Cell Culture, synchronization and transfection

HEK293T (ACC 635, DSMZ Braunschweig, Germany), HeLa (ATCC CCL-2) (David Beach, Cold Spring Harbor) and U2OS (ATCC HTB-96) (A. Fry/E. Nigg, Basel) cells were cultured in DMEM containing 4.5 g/l glucose supplemented with 10% fetal bovine serum (Sigma), 2 mM L-glutamine (Sigma), 100 U/ml penicillin and 0.1 mg/ml streptomycin (Sigma) at 37°C in 5% CO_2_. Cell line authentication was performed by Multiplexion, Heidelberg. For double thymidine block of HeLa cells, cells were treated with 4 mM thymidine for 16 h, released for 8 h, and again blocked for 16 h. To arrest U2OS cells in prometaphase, cells were first synchronized in S phase with 1.6 µg/ml aphidicolin (Sigma) for 17 h, released for 5 h, and again blocked with 100 ng/ml nocodazole (AppliChem) for 16 h. HEK293T cells were transfected for 24 h with polyethylenimine (Polysciences, Inc.) at a final concentration of 5 µg/ml using 27 µg plasmid DNA per 15 cm dish (2×10^7^ cells). U2OS cells were transfected using polyethylenimine or Lipofectamine 2000 (Invitrogen) according to the manufacturer's instructions.

### Plasmid constructions

STIL cDNA was amplified by PCR from pENTR22.3-STIL (GenBank accession number BC126223.1, obtained from Genomics and Proteomics Core Facility/S. Wiemann, DKFZ Heidelberg, Germany) and cloned into the SalI and XhoI sites of pCMV-3Tag1A (Agilent Technologies) and into the XhoI and ApaI sites of pEGFP-C3. The STIL fragments were amplified by PCR and cloned into pCMV-3Tag1A (STIL 1-231/231-619/619-781 by SalI/ApaI, STIL 781-1287 by BamHI/XhoI, STIL 1-619/1-781 by EcoRI/SalI, STIL 619-1287 by HindIII/XhoI) or into pGEX-4T1 (STIL 1-619 by EcoRI/SalI and STIL 619-1287 by SalI/NotI). pCMV-3Tag1A-Plk4 full length and fragments, pCMV-3Tag2A-Plk4, pQE80zz-Plk4 and pMAL-c2-Plk4 have been described previously (Cizmecioglu et al., 2010).

Mutations were introduced by PCR-based site-directed mutagenesis (Quik Change Lightning Multi-Site directed Mutagenesis Kit, Agilent) using pCMV-3Tag-1A-STIL as a template.

### Antibodies

Mouse anti-Plk4 antibody has been described previously (Cizmecioglu et al., 2010) and used at a final concentration of 1 µg/ml. Mouse anti-Flag M2 (F3165), mouse anti-α-tubulin (T5168) and mouse anti-γ-tubulin (T6557) were from Sigma. Mouse anti-Myc (9E10), mouse anti-Plk1 (F-8), and mouse anti-cyclin E (HE12) were obtained from Santa Cruz Biotechnology. Mouse anti-actin (JLA20) was from Calbiochem, mouse anti-His from Qiagen, rabbit anti-GFP (NB600-303) from Novus, rabbit anti-CP110 (A301-343A) and rabbit anti-STIL (A302-442A) for western blotting from Bethyl, rabbit anti-STIL (ab89314) for immunofluorescence and rabbit anti-pericentrin (ab4448) from Abcam. Rabbit anti-cyclin B1 has been described previously (Hoffmann et al., 1993). Secondary antibodies for western blotting were peroxidase-conjugated donkey anti-rabbit (Jackson Laboratories) and goat anti-mouse (Novus). Secondary antibodies for immunofluorescence were goat anti-mouse IgG and goat anti-rabbit IgG coupled to Alexa Fluor 488 or Alexa Fluor 594 (Molecular Probes).

### Western blotting, immunoprecipitation and pull down assay

Cell lysates were prepared with NP40 lysis buffer (40 mM Tris pH 7.5, 150 mM NaCl, 0.5% NP40, 5 mM EDTA, 10 mM β-glycerophosphate, 5 mM NaF, 1 mM DTT, 0.1 mM Na_3_VO_4_ and protease inhibitors). For pull down of Zz-Plk4 from HeLa lysates, 3 mg of cell lysate was incubated with 15 µg of Zz-Plk4 for 2 h at 4°C, followed by addition of rabbit IgG Sepharose for 1 h at 4°C. For immunoprecipitations, 2–6 mg of cell lysates were incubated for 2–4 h or over night at 4°C with anti-Flag M2 affinity beads (Sigma) or 2–8 µg anti-Myc, anti-Plk4 or anti-STIL antibodies and normal mouse or rabbit IgG as control, respectively, followed by addition of 15 µl Protein G or A Sepharose (GE Healthcare). Beads were washed with NP40 buffer three or four times, eluted by incubation with sample buffer for 10 min at room temperature or by competition with 500 ng/µl 3× Flag peptide, boiled in sample buffer and analyzed by SDS-PAGE and western blotting, performed according to a standard protocol (Hassepass et al., 2003). Immunoreactive signals were detected with Immobilon Western Chemiluminescent HRP substrate (Millipore).

### Recombinant protein expression and *in vitro* kinase assay

Expression of Zz–Plk4-His has been described previously (Cizmecioglu et al., 2010; Jäkel and Görlich, 1998). GST–STIL fragments were expressed in *E. coli* BL21-Rosetta and natively purified by single-step affinity chromatography using glutathione-agarose according to the manufacturer's protocol. For *in vitro* kinase assays with recombinant Zz-Plk4-His, Flag-STIL FL or fragments were expressed in HEK293T cells and immunoprecipitated using anti-Flag M2 affinity beads (Sigma) as described above. Flag-STIL bound to agarose beads was washed three times in NP40 buffer and once in kinase buffer (50 mM Tris pH 7.5, 10 mM MgCl_2_, 10 µM MnCl_2_, 1 mM DTT) followed by an incubation with 2.5–5 µg Zz–Plk4-His in the presence of 5 µCi [γ-^32^P]-ATP (PerkinElmer) in kinase assay buffer supplemented with 33 µM ATP for 15–20 min at 30°C. Reactions were stopped by adding sample buffer, elution for 10 min at RT and heating at 95°C. Samples were analyzed by SDS-PAGE followed by Coomassie Blue staining and autoradiography.

### Indirect immunofluorescence microscopy

For indirect immunofluorescence, cells grown on coverslips were fixed with −20°C methanol for 10 min. Afterwards, cells were permeabilized with PBS/0.05% Triton X-100 (PBSX) for 10 min, washed with PBS and blocked with 3% BSA in PBSX for 30 min. Cells were incubated with primary antibodies diluted in 3% BSA/PBSX for 1 h, then washed three times with PBSX and incubated with secondary antibodies and 1 µg/ml Hoechst 33258 (Invitrogen) for 1 h. After washing three times with PBSX, coverslips were mounted onto glass slides with ProLong Gold (Molecular Probes). For cell imaging, the Zeiss motorized inverted Observer.Z1 microscope was used, containing mercury arc burner HXP 120 C and LED module Colibri. Filter combinations: GFP (38 HE) DsRed (43 HE) and DAPI (49) with the detector gray scale CCD camera AxioCam MRm system and a 63×/1.4 Oil Pln Apo DICII objective. Image processing was performed using Fiji software.

### Mass spectrometry analysis

For identification of Zz-Plk4-interacting proteins, Zz-Plk4 elution fractions were resolved by SDS-PAGE and coprecipitating proteins were detected in gel by staining with Colloidal Coomassie. Analysis was performed at the DKFZ Protein Analysis Facility (Heidelberg, Germany). The gel lanes were cut into slices, digested with trypsin after reduction and alkylation of cysteines. Tryptic peptides were analyzed by nanoLC-ESI-MS/MS using a nanoAcquity UPLC system (Waters GmbH) coupled online to an LTQ Orbitrap XL mass spectrometer (Thermo Scientific). Data were acquired by scan cycles of one FTMS scan with a resolution of 60,000 at m/z 400 and a range from 300 to 2000 m/z in parallel with six MS/MS scans in the ion trap of the most abundant precursor ions. Instrument control, data acquisition and peak integration were performed using the Xcalibur software 2.1 (Thermo Scientific, Bremen, Germany).

Database searches were performed against the SwissProt database with taxonomy “human” using the MASCOT search engine (Matrix Science, London, UK; version 2.2.2). MS/MS files from the individual gel slices of each lane were merged into a single search. Peptide mass tolerance for database searches was set to 5 ppm and fragment mass tolerance was set to 0.4 Da. Significance threshold was p<0.01. Carbamidomethylation of cysteine was set as fixed modification. Variable modifications included oxidation of methionine and deamidation of asparagine and glutamine. One missed cleavage site in case of incomplete trypsin hydrolysis was allowed.

For identification of Plk4-phosphorylated residues of STIL, bacterially purified GST-tagged STIL fragments were phosphorylated by Zz-Plk4 in an *in vitro* kinase assay as described above, resolved by SDS-PAGE and stained with Colloidal Coomassie. Mass spectrometry was performed at the ZMBH Core facility for mass spectrometry and proteomics (Heidelberg). STIL protein bands were excised, reduced with DTT, alkylated with iodoacetamide and digested with trypsin or GluC using a Digest pro MS liquid handling system (Intavis AG), as described previously (Catrein et al., 2005). Digested peptides were analyzed by a nanoHPLC system coupled to an Orbitrap XL mass spectrometer (Thermo Fisher Scientific).

## RESULTS

### Plk4 interaction with STIL

**Fig. 1.**
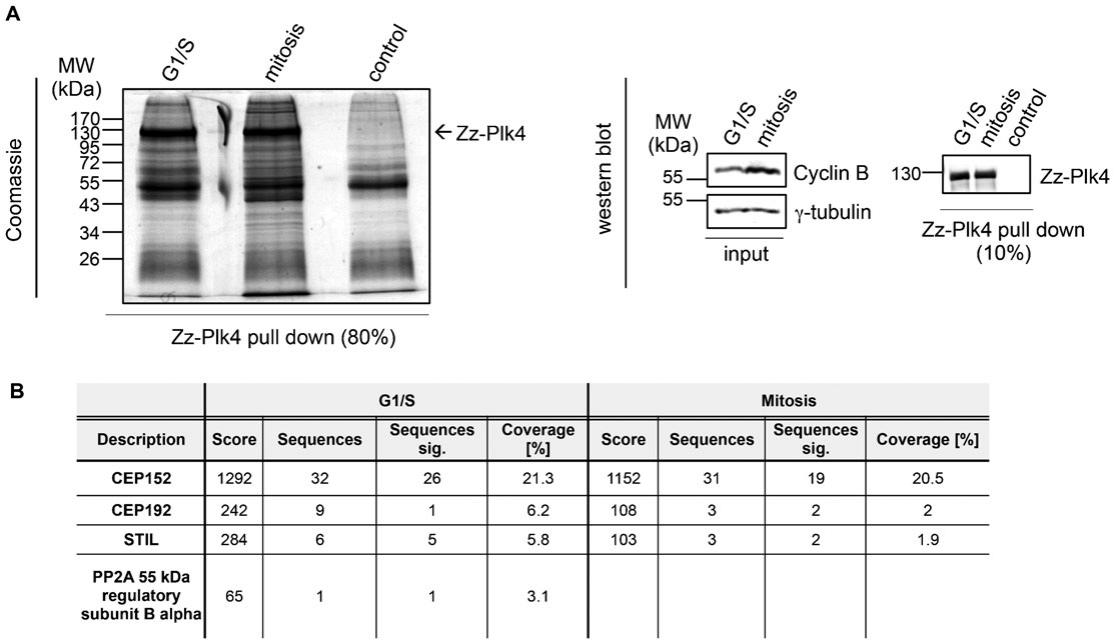
Identification of STIL as a Plk4 interacting protein. (A) Bacterially purified Zz-Plk4 (N-terminal Zz-tag and C-terminal His-tag) was incubated with G1/S or mitotically arrested (double thymidine block and release) HeLa Kyoto cell lysates. Cell lysates alone served as control. Plk4 was immunoprecipitated via its Zz-tag and eluted with its interaction partners. Coimmunoprecipitating proteins were detected by staining with Colloidal Coomassie (left panel) and analyzed by mass spectrometry. Western blotting (right panel) using anti-His antibodies was performed to detect Zz-Plk4 by its His-tag in elution fractions. Cyclin B abundance was used to determine the cell cycle stages of the lysates and anti γ tubulin detection served as a loading control. (B) Mass spectrometry analysis of Zz-Plk4 pull down identified known Plk4 interaction partners, substrates or regulators and STIL as a novel Plk4 interaction partner.

**Fig. 2.**
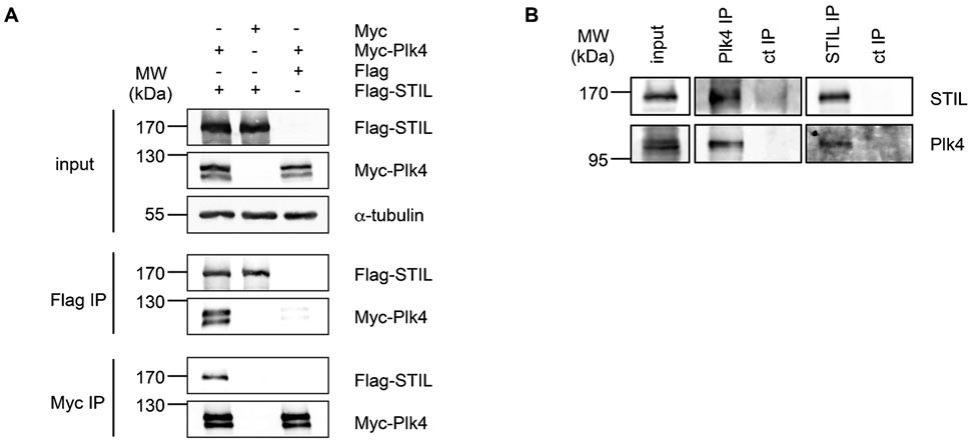
STIL interacts with Plk4 *in vivo*. (A) Coimmunoprecipitation of Flag-STIL and Myc-Plk4. Lysates from HEK293T cells transfected with the indicated plasmids were subjected to immunoprecipitations using anti-Flag or anti-Myc antibodies. Input and IP samples were analyzed by western blotting with antibodies against Flag-tag or Plk4 and α-tubulin as loading control. (B) Coimmunoprecipitation of endogenous STIL and Plk4. Endogenous Plk4 and STIL were immunoprecipitated from HeLa lysates by anti-Plk4 and anti-STIL antibodies. IP with non-specific IgGs served as a control. Coimmunoprecipitated proteins were detected by western blotting using anti-Plk4 and anti-STIL antibodies.

During a project aimed at identifying novel binding partners and substrates of Plk4, a major regulator of centriole duplication, we performed a biochemical pull down assay with recombinant double-tagged Plk4 (N-terminal Zz-tag, which consists of two IgG binding domains from protein A and C-terminal His-tag) with extracts derived from HeLa cells (Fig. 1A). Mass spectrometry analysis of eluted binding partners identified STIL, a centriole duplication factor that localizes to the cartwheel in procentrioles. Apart from STIL, a number of known Plk4-interacting proteins, among them Cep152 (Cizmecioglu et al., 2010; Dzhindzhev et al., 2010; Hatch et al., 2010), Cep192 (Sonnen et al., 2013) and a subunit of PP2A (Brownlee et al., 2011), were identified (Fig. 1B). We then confirmed this interaction by using Flag-STIL and Myc-Plk4 overexpression in HEK293T cells followed by reciprocal immunoprecipitations (Fig. 2A). To characterize this interaction in more detail, we analyzed whether complexes between endogenous STIL and Plk4 could be detected *in vivo*. As seen in Fig. 2B, endogenous STIL was identified in Plk4 immunoprecipitates and vice versa endogenous Plk4 was detected in immunoprecipitation experiments using antibodies against STIL. These results demonstrate that STIL and Plk4 form a complex *in vivo* confirming our initial findings based on mass spectrometry.

**Fig. 3.**
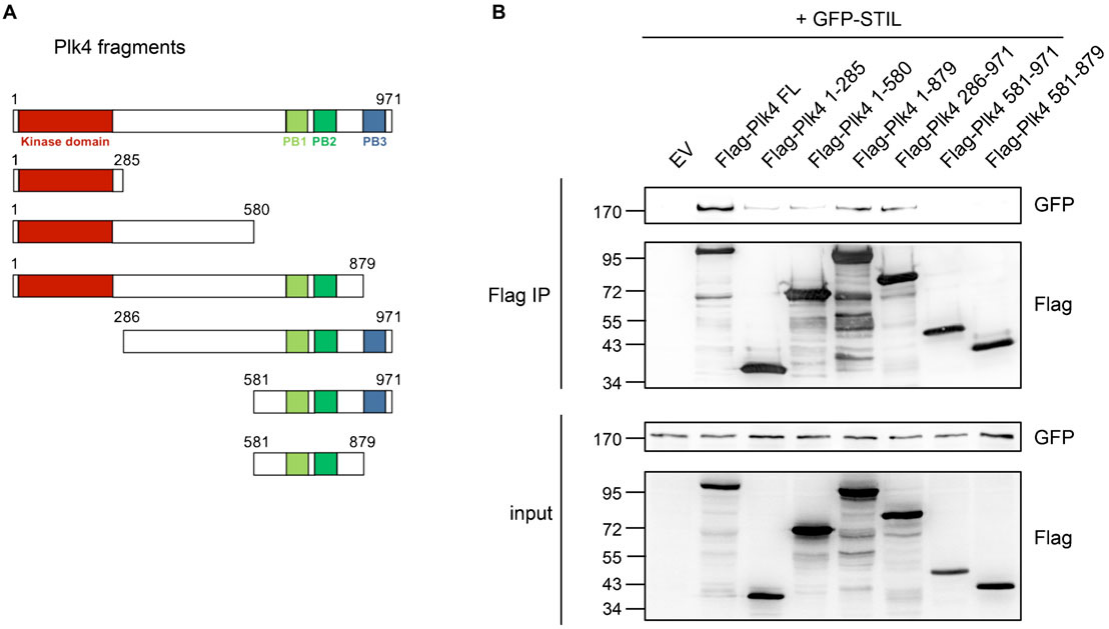
The polo-boxes of Plk4 are not sufficient to mediate STIL binding. (A) Scheme of Flag-Plk4 fragments. (B) After overexpression of Flag-Plk4-fragments (A) and GFP-STIL in HEK293T cells, cell lysates were subjected to immunoprecipitations using anti-Flag antibodies. Coprecipitation of GFP-STIL with Flag-Plk4 fragments was detected by western blotting using anti-GFP and anti-Flag antibodies.

**Fig. 4.**
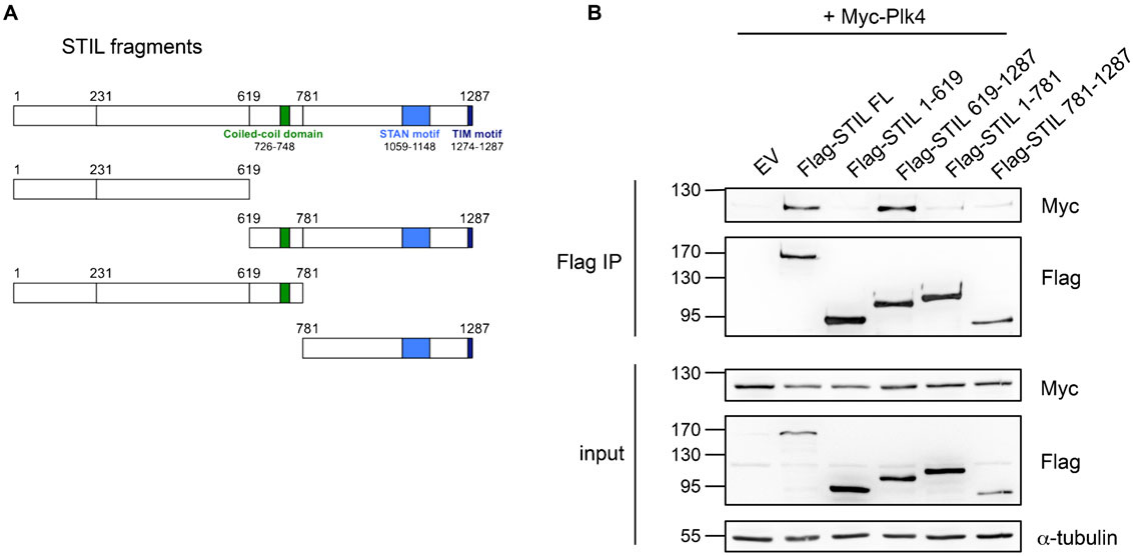
The C-terminus of STIL interacts with Plk4. (A) Scheme of Flag-STIL fragments. (B) After overexpression of Flag-STIL-fragments (A) and Myc-Plk4 in HEK293T cells, cell lysates were subjected to immunoprecipitations using anti-Flag antibodies. Coprecipitation of Myc-Plk4 with Flag-STIL fragments was detected by western blotting using anti-Myc and anti-Flag antibodies.

Plk4 is a structurally divergent Plk family member. The Plk4 sequence contains an amino-terminal kinase domain as well as a tandem homodimerized polo-box domain (PB1-PB2) and a C-terminal polo-box (PB3) (Slevin et al., 2012). To investigate the interaction between Plk4 and STIL in more detail, we mapped the binding sites between Plk4 and STIL. For the Plk4 interacting protein Cep152 at the centrosome (Cizmecioglu et al., 2010; Dzhindzhev et al., 2010; Hatch et al., 2010) it was shown that the two tandem polo-boxes PB1 and PB2 of Plk4 are required for binding. Interestingly, using the same Plk4 fragments as described in (Cizmecioglu et al., 2010) to show the interaction between Cep152 and Plk4 (Fig. 3A), we observed that neither PB1, 2 or 3 are sufficient to mediate binding of Plk4 to STIL (Fig. 3B). To further map the interaction between the two proteins, we also generated truncated STIL fragments (Fig. 4A). Coexpression of full-length Plk4 with STIL fragments in HEK293T cells followed by immunoprecipitation using antibodies against the Flag-tag showed that STIL interacted strongest with Plk4 when the C-terminal fragment 619–1287 was expressed. This fragment harbors both the coiled-coil domain and the STAN motif of STIL (Fig. 4B).

### STIL and Plk4 colocalize at centrioles at the onset of S phase

**Fig. 5.**
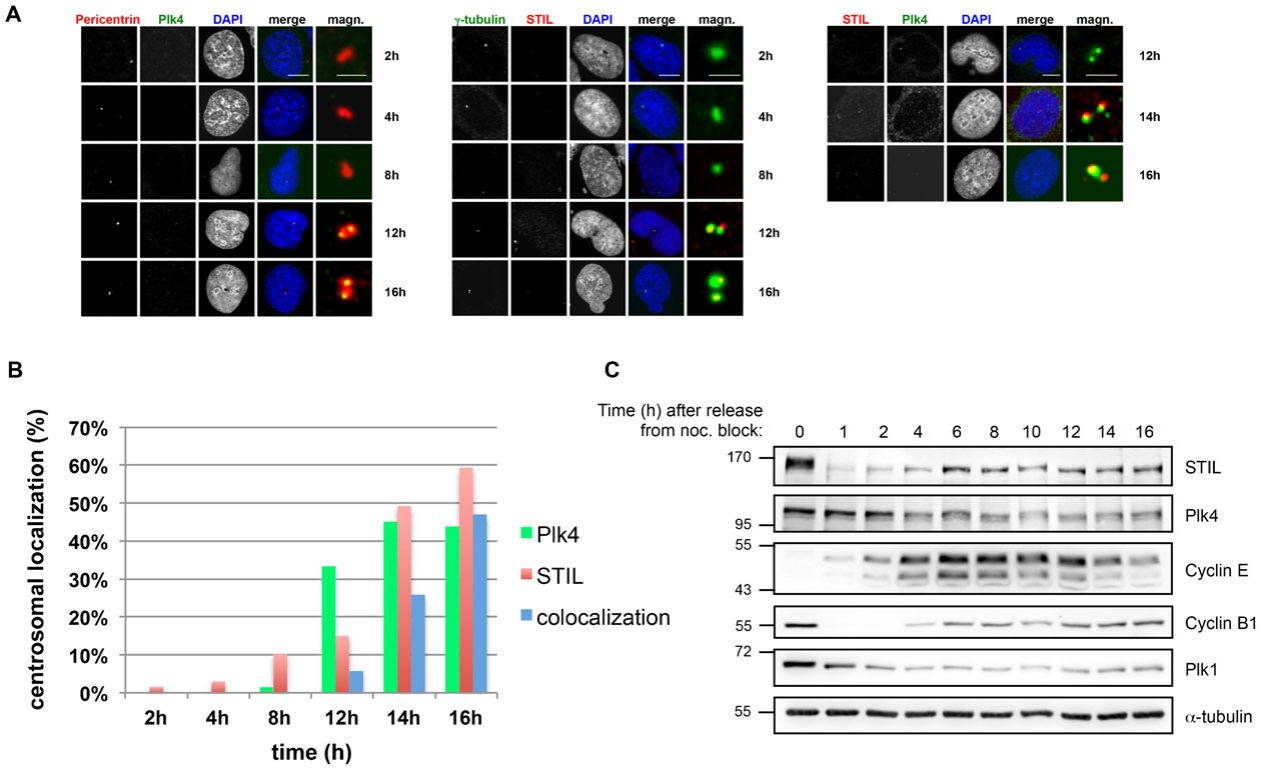
Colocalization of STIL and Plk4 at the onset of S phase. (A–C) U2OS cells were arrested in S-phase with 1.6 µg/ml aphidicolin for 17 h, released for 5 h and then rearrested in prometaphase by addition of 100 ng/ml nocodazole for additional 16 h. The cells were released and harvested at the indicated time points. (A) For indirect immunofluorescence analysis, cells were fixed and stained with antibodies against Plk4, pericentrin, STIL and γ-tubulin. Scale bars: 10 µm (merge), 2 µm (magnifications). (B) Centrosomal localization of Plk4 and STIL was quantified for the indicated time points by counting colocalization with the centrosomal marker proteins γ-tubulin and pericentrin. For each time point on average 60 cells were counted. (C) Synchronization of the cells was confirmed by western blot analysis using antibodies against Plk1, Plk4, Cyclin E, Cyclin B1, STIL, and α-tubulin.

We then investigated the colocalization of Plk4 with STIL at the onset of centriole duplication around the G1/S phase transition. U2OS cells were synchronized in prometaphase using nocodazole and then released from the block. Samples were taken at the indicated time points and localization of both STIL and Plk4 at the centrosome was determined (Fig. 5A). Plk4 is not present at centrioles during late mitosis and early G1 phase but then accumulates at centrioles around 12 h after release from the nocodazole block. We and others found, similar to Plk4, that STIL is not detected at centrioles during late mitosis (Arquint et al., 2012) but starts to localize to the centriole in late G1 phase. At 14 h after release from the block both proteins colocalize at a time point which coincides with the onset of S phase entry and centriole duplication Fig. 5B. As excess of STIL triggers centriole amplification, we also investigated how the protein expression levels of STIL correlated with those of Plk4 in the same experiment as shown in Fig. 5A. As previously demonstrated (Tang et al., 2011; Arquint et al., 2012), STIL levels were high during metaphase but then dropped abruptly (similar to cyclin B degradation) at the exit from mitosis. STIL levels then rise again during mid-G1 phase. On the contrary, Plk4 protein expression was detected during both metaphase and G1/S phase with a slight increase in protein levels in metaphase and early G1 phase even though it was not present on centrioles at the exit of mitosis (Fig. 5C).

### Phosphorylation of STIL by Plk4 triggers centriole duplication

**Fig. 6.**
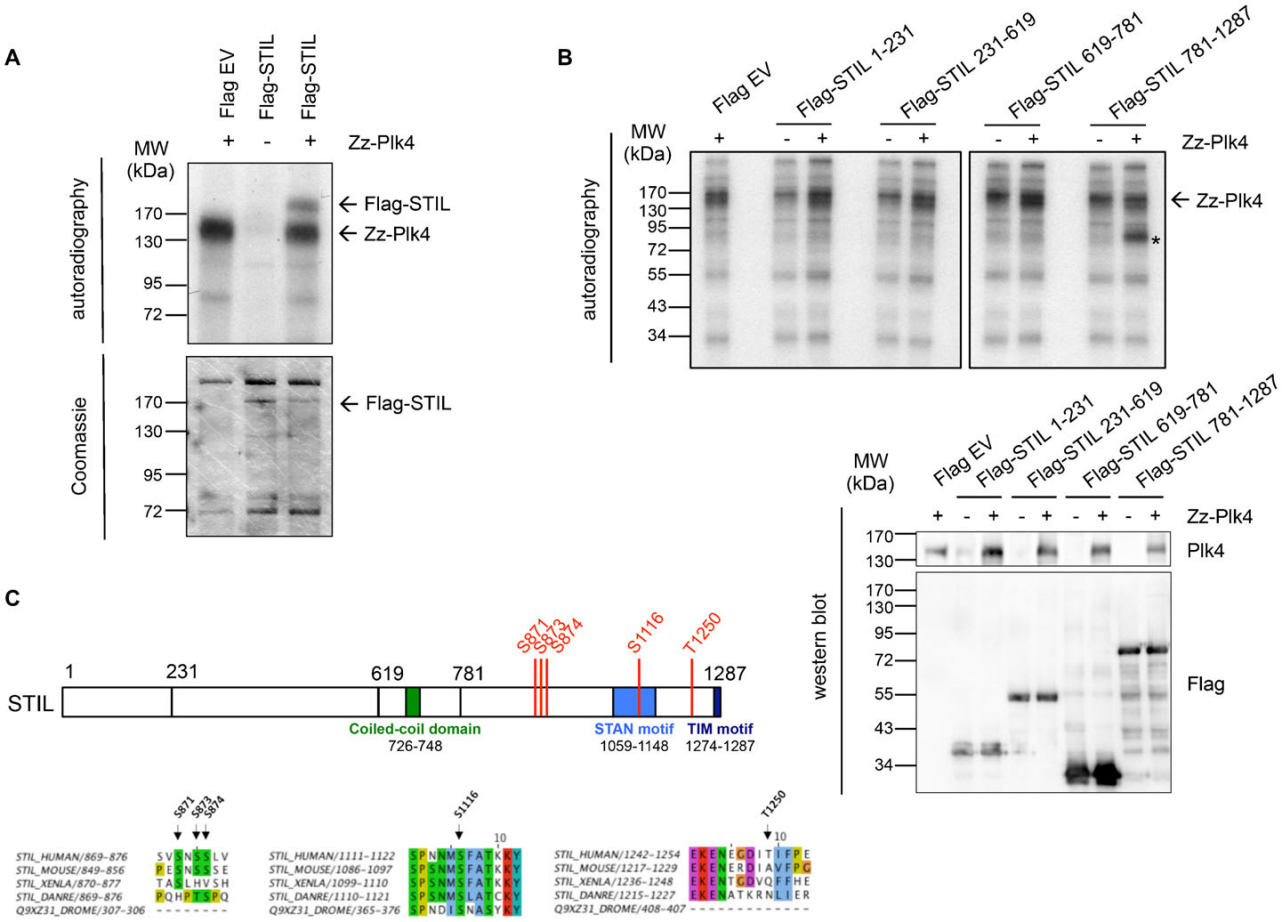
Phosphorylation of STIL by Plk4. (A) Full-length Flag-STIL expressed in HEK293T cells and immunoprecipitated with anti-Flag antibodies was incubated with bacterially expressed Zz-Plk4 in the presence of [γ-^32^P]-ATP. *In vitro* kinase assay with Flag-STIL or Plk4 alone served as a control. Kinase assays were analyzed by SDS-PAGE, Coomassie Blue staining and autoradiography. (B) Indicated Flag-STIL fragments were expressed in HEK293T cells and immunoprecipitated with anti-Flag antibodies. Immunoprecipitation fractions were incubated with bacterially expressed Zz-Plk4 in the presence of [γ-^32^P]-ATP, followed by SDS-PAGE and autoradiography. *In vitro* kinase assay with Flag-STIL fragments or Plk4 alone is shown as control. The asterisk indicates phosphorylated Flag-STIL 781-1287. 10% of each precipitation fraction was analyzed by western blotting using anti-Plk4 and anti-Flag antibodies. (C) Plk4 phosphorylation sites in the STIL protein identified by mass spectrometry analysis of bacterially purified GST-STIL 1-619 and 619-1287 phosphorylated *in vitro* by Zz-Plk4. Alignment of the identified sites in human, mouse, *Xenopus* and zebrafish STIL and *Drosophila* Ana2 is shown.

To explore the possibility that STIL is directly phosphorylated by Plk4, we performed *in vitro* kinase assays. A Zz-tagged kinase active form of Plk4 was shown to phosphorylate immunoprecipitated Flag-tagged STIL (Fig. 6A).

To narrow down the region within STIL that is phosphorylated by Plk4 we expressed truncated versions of Flag-tagged STIL in cells and performed *in vitro* kinase assays again using Zz-tagged Plk4 wt. We found that a C-terminal fragment of STIL containing the STAN motif was heavily phosphorylated (Fig. 6B). To identify Plk4-specific phosphorylation sites, we subjected *in vitro* phosphorylated STIL N- and C-terminal fragments to mass spectrometry and identified five Plk4-specific phosphorylation sites on serine and threonine residues at the C-terminal domain of STIL, namely S871, S873, S874, S1116 and T1250. Interestingly, S1116 is located in the STAN motif of STIL and conserved among vertebrates and in *Drosophila* (Fig. 6C).

**Fig. 7.**
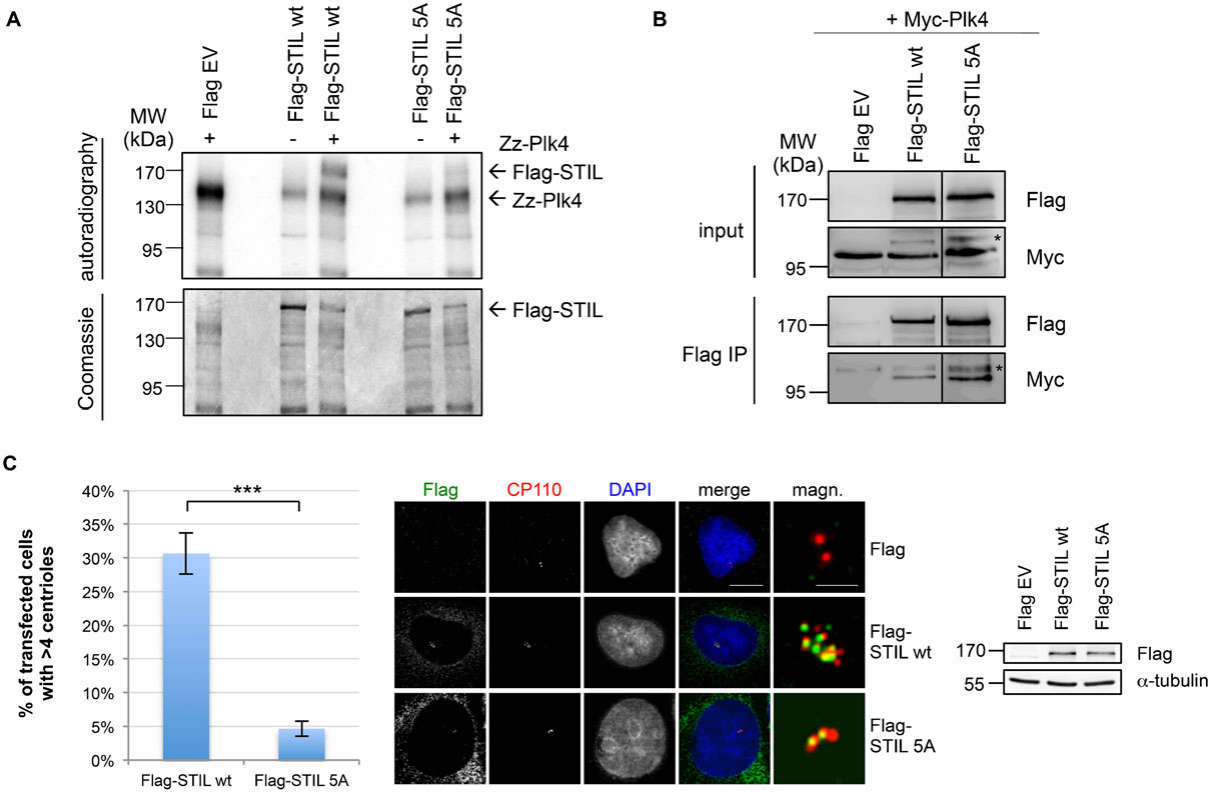
Phosphorylation of STIL by Plk4 triggers centriole duplication. (A) Flag-STIL full-length or 5A mutant (S871A/S873A/S874A/S1116A/T1250A) expressed in HEK293T cells and immunoprecipitated with anti-Flag antibodies was incubated with bacterially expressed Zz-Plk4 in the presence of [γ-^32^P]-ATP. *In vitro* kinase assay with Flag-STIL or Plk4 alone served as a control. Kinase assays were analyzed by SDS-PAGE, Coomassie Blue staining and autoradiography. (B) Co immunoprecipitation of Flag-STIL wt/5A and Myc-Plk4. Lysates from HEK293T cells transfected with the indicated plasmids were subjected to immunoprecipitation using anti-Flag antibodies. Input and IP samples were analyzed by western blotting with antibodies against Flag- and Myc-tag. The asterisk marks an unspecific band recognized by the anti-Myc antibody. The dividing lane indicates grouping of images from different parts of the same gel, as an intervening lane was removed for presentation purposes. (C) U2OS cells transiently expressing Flag EV, Flag-STIL wt or Flag-STIL 5A were analyzed by indirect immunofluorescence using staining with anti-CP110 and mouse anti-Flag antibodies 72 h after transfection. The number of transfected cells with more than four centrioles was determined based on CP110 staining. Values in the graph are mean percentages±s.d. from three independent experiments, 50 transfected cells were analyzed in each experiment (***P<0.001, two-tailed t-test). Representative images are shown for control, Flag-STIL wt and 5A-transfected cells. Scale bars: 10 µm (merge), 2 µm (magnifications). Western blotting using antibodies against Flag and α-tubulin was performed to visualize expression of STIL constructs as indicated.

We then asked whether phosphorylation of STIL by Plk4 contributes to its function in centriole duplication. We mutated the five phosphorylation sites into non-phosphorylatable alanine residues and showed that this leads to a reduction in the incorporation of phosphate in comparison to wild-type STIL in an *in vitro* kinase assay again using recombinant active Plk4 (Fig. 7A). Interestingly, Flag-STIL wt and Flag-STIL 5A were bound in equal amounts to Plk4 (Fig. 7B). To explore the impact of the phosphorylation sites on centriole duplication, we first verified that the mutated STIL 5A showed a centriolar localization similar to STIL wt (Fig. 7C, right panel). Taken together, these results suggested that the phosphorylation of STIL by Plk4 is not required for the interaction of the two proteins and the correct localization of STIL. To further explore the impact of the Plk4-dependent phosphorylations on STIL function, we tested the capability of both STIL wt and STIL 5A to overduplicate centrioles. Upon overexpression of Flag-STIL wt in U2OS cells, we found that around 30% of transfected cells exhibited a centriole overduplication phenotype (cells >4 centrioles), while expression of Flag-STIL 5A failed to promote centriole overduplication.

Together, these results show that STIL is a novel Plk4 interacting protein and substrate and that Plk4-dependent phosphorylation of STIL triggers centriole duplication.

## DISCUSSION

Studies in the nematode *Caenorhabditis elegans* describe the initial stage of centriole assembly (Strnad and Gönczy, 2008). Spd2 (Cep192) is required for the centriolar localization of Zyg1, an ortholog of human Plk4. Subsequently, both proteins then promote the assembly of a complex of the two coiled-coil proteins Sas-5 (STIL) and Sas-6 to the procentriole, thus triggering the formation and elongation of the centriolar tube. Sas-4 contributes to the assembly of microtubules resulting in the formation of a new centriole (Pelletier et al., 2006; Delattre et al., 2006). The Sas-6-Sas-5 complex is recruited to the site of procentriole formation immediately after Zyg1 recruitment, but it seems that Sas-6 is an unlikely target of Zyg1 (Lettman et al., 2013). While this study was in progress two papers appeared showing that phosphorylation of STIL by Plk4 triggers Sas-6 recruitment to promote procentriole formation (Ohta et al., 2014; Dzhindzhev et al., 2014). While a previous study (Ohta et al., 2014) indicates that an interaction between STIL and Plk4 requires the two tandem polo-boxes, PB1 and PB2, our own findings imply that these domains are not sufficient for binding to STIL and that in particular the N-terminus of Plk4 is required for STIL binding. In Fig. 4B we show that the region harboring the PB1 and PB2 which has previously been shown to bind to Cep152 (Cizmecioglu et al., 2010) fails to bind STIL. Ohta et al., 2014 demonstrated a reduced binding of STIL to Plk4 when PB1 or PB2 domains were deleted. However, it remains unclear from their study whether a fragment comprising PB1 and PB2 is able to bind to STIL. Our findings therefore suggest that Plk4 harbors a so far undiscovered substrate binding domain that is located between the catalytic domain and the polo-box domain.

STIL is translocated from the centrosome to the cytoplasm and then degraded in early anaphase by the APC/C (Arquint and Nigg, 2014). Our data confirm this observation and further show that STIL and Plk4 together relocalize to the centrosome during mid G1 and S phase (Fig. 5) which coincides with the onset of centriole duplication. Interestingly, in contrast to STIL, cellular Plk4 levels do not significantly drop upon exit from mitosis, whereas, similar to STIL, Plk4 is released from centrioles at this time of the cell cycle. This observation implicates that Plk4 also exerts another function independent from its localization to the centrioles.

A particular evolutionary conserved region within the C-terminus of Ana2 and STIL has been denoted the STil/ANa2 (STAN) motif (Stevens et al., 2010a). Experiments using truncations of the STAN motif have shown that it is required for centrosomal localization and duplication (Vulprecht et al., 2012; Arquint and Nigg, 2014). Ohta et al. (Ohta et al., 2014) identified the STIL coiled-coil (CC) domain to be required for binding to Plk4 and observed that binding to Plk4 was unaffected when the STAN motif was deleted. We find that a fragment harboring both CC domain and STAN motif is required for Plk4 binding. However, as we observe a weak binding in a CoIP between Plk4 and the STIL 1-781 (CC) and the STIL 781-1287 (STAN) constructs (Fig. 4B) it is plausible that either domain (CC or STAN) may weakly bind in isolation but collaborate to achieve a robust binding. Additionally, we could show that STIL is directly phosphorylated by Plk4. We identified five serine/threonine residues in the C-terminus of STIL to be phosphorylated by Plk4. These include a phosphorylation at S1116, which is located within the STAN domain and highly conserved in both vertebrates and *Drosophila* (Fig. 6C). Overexpression of a non-phosphorylatable STIL 5A mutant failed to promote centriole overduplication, but did not impair the binding of STIL to Plk4 and centriolar localization of STIL (Fig. 7). This indicated that phosphorylation of STIL by Plk4 may not be essential for the interaction of STIL and Plk4 and for the localization of STIL at the centrioles in human cells, but required to trigger centriole duplication. As the STAN domain has been implicated in centriole duplication, we speculate that in particular phosphorylation on S1116 is involved in centrosome amplification. Interestingly, in previous studies (Ohta et al., 2014; Dzhindzhev et al., 2014), phosphorylation of S1116 (S370 in *Drosophila melanogaster*) in STIL was also observed and shown to be important for centriole duplication.

Future studies will be required to demonstrate how and when during the early cell cycle stages phosphorylation of STIL by Plk4 will initiate procentriole formation.
